# Global Analysis of Cell Type-Specific Gene Expression

**DOI:** 10.1002/cfg.281

**Published:** 2003-04

**Authors:** David W. Galbraith

**Affiliations:** University of Arizona Department of Plant Sciences 303 Forbes Building Tucson AZ 85721 USA

## Abstract

The tissues and organs of multicellular eukaryotes are frequently observed to comprise complex three-dimensional interspersions of different cell types. It is a reasonable assumption that different global patterns of gene expression are found within these different cell types. This review outlines general experimental strategies designed to characterize these global gene expression patterns, based on a combination of methods of transgenic fluorescent protein (FP) expression and targeting, of flow cytometry and sorting and of high-throughput gene expression analysis.


**Comparative and Functional Genomics**
*Comp Funct Genom* 2003; **4**: 208–215.
Published online 1 April 2003 in Wiley InterScience (www.interscience.wiley.com). **DOI:** 10.1002/cfg.281

**Conference Review**

### Global analysis of cell type-specific gene expression

David W. Galbraith*
*University of Arizona, Department of Plant Sciences, 303 Forbes Building, Tucson, AZ 85721, USA*

*Correspondence to:
David W. Galbraith, University of Arizona, Department of Plant Sciences, 303 Forbes Building, Tucson, AZ 85721, USA.
E-mail: galbraith@arizona.edu*

Received: 4 February 2003
Revised: 5 February 2003
Accepted: 6 February 2003

### Abstract

**The tissues and organs of multicellular eukaryotes are frequently observed to comprise complex three-dimensional interspersions of different cell types. It is a reasonable assumption that different global patterns of gene expression are found within these different cell types. This review outlines general experimental strategies designed to characterize these global gene expression patterns, based on a combination of methods of transgenic fluorescent protein (FP) expression and targeting, of flow cytometry and sorting and of high-throughput gene expression analysis. Copyright © 2003 John Wiley & Sons, Ltd.**

**Keywords:** **gene expression; GFP; flow cytometry and sorting; eukaryotes; microarrays; high-throughput methods**

### Introduction

With the advent of techniques for high-throughput analysis of gene expression, interest is arising in the possibility that these methods could be applied to categorize gene expression, not simply in organs and tissues but also within the different individual cell types found in living organisms. Recent estimates suggest that there are 200–300 different cell types within most mammalian organs, with possibly as many as 1000 within the brain [2]. Clearly, the ability to chart differences and similarities in gene expression patterns within these different cell types would provide considerable insight into the mechanisms that govern their development, function and responses to the environment.

The term 'gene expression' at its most general can be defined as linking the genotype of an organism to a corresponding phenotype. However, the latter is an inextricable function of the selected method for empirical observation, be it morphology, biochemical assay or high-throughput platform. Mechanistically, gene expression is a complex, regulated process, conventionally regarded as starting with the information content of the genome and linked ultimately to the molecules that directly implement the phenotype that is being measured. Dissection of the process of gene expression into its respective mechanistic components clearly should provide deeper insight into its means of function than would e.g. observation of Mendelian segregation of a locus governing a visible phenotype.

This review provides a brief survey of established and emerging methods for experimental analysis of global gene expression, focusing on high-throughput platforms, and then goes on to consider how these might be applied for the analysis of global gene expression within specific cell types, using higher plant systems as the primary example.

### Platforms for global gene expression analysis

At the molecular level, the most widely employed high-throughput methods concern analysis of mRNA levels. These include various methods that can be grouped according to whether they are based on immobilized, high-density arrays of DNA (microarrays [31], GeneChips [29], ArrayPlates [24], Agilix [**http://www.agilixcorp.com]**) or based on signature sequencing (SAGE [35];

Copyright © 2003 John Wiley & Sons, Ltd.

Lynx-MPSS [3,4]) and various methods involving use of diagnostic cDNA sequence-length polymorphisms [1,25,36]. These methods were developed largely as a consequence of the ease with which DNA can be experimentally manipulated, reflecting the fact that differences between individual DNA molecules are encoded within the base sequences and do not result in differences in physicochemical properties of the molecule. The methods centre on the global analysis of one class of polynucleotide macromolecule, polyadenylated RNA, and thereby provide information about gene expression as reflected in the steady state intracellular concentrations of mRNA within the tissues or organs of interest. As indicated in Figure 1, accumulation of cytoplasmic mRNA is a combination of processes of intranuclear synthesis and processing and nuclear export, and cytoplasmic degradation. Analysis of cytoplasmic mRNA by itself clearly cannot discriminate between the contributions of these two processes. We have proposed [25] an examination of the polyA$^+$ RNA content of nuclei as a means to insert greater precision into the analysis of gene expression, involving a focus on the initial process of transcription and intranuclear message processing. Other published approaches have included selectively examining only those mRNA molecules that are being translated [5,22], and these similarly introduce greater precision into the analysis of the overall process of gene expression.

The available experimental platforms for analysis of global gene expression can be ranked according to the specificity with which they report the contributions of individual genes. Classical microarrays, produced from PCR amplicons of known or unknown sequence, are subject to cross-hybridization between domains of similar sequence. Empirically, cross-hybridization is readily detected when two DNA molecules exceed 70% sequence identity [8,39]. Cross-hybridization also can occur if genes of otherwise dissimilar sequence contain regions of identity exceeding around 20 bp in length [39]. Problems of cross-hybridization can be ameliorated, and ideally eliminated, by use of long, presynthesized oligonucleotides (50- to 70-mers) as microarray elements. Assuming sufficient genomic sequence is available, these can be designed in such a manner as to be unique to the gene of interest. Other criteria for sequence design

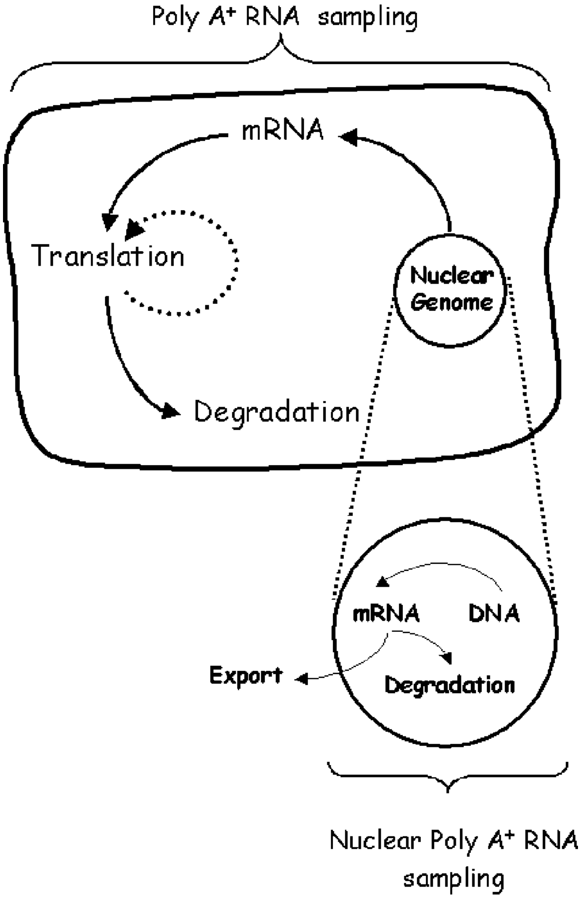

**Figure 1.** Conventional polyA$^+$ RNA sampling for gene expression analysis provides a measure of the steady-state concentrations within the cell, whereas sampling of purified nuclei focuses attention more closely on transcriptional events include positioning the sequence within a reasonable distance upstream of the 3′ end of the transcript, selecting sequences that have similar melting temperatures, and eliminating from consideration those capable of adopting stem–loop configurations of greater than a certain degree of stability. Applying these criteria stringently can eliminate from consideration, for specific genes, all available sequences, in which case one or more of the criteria must be relaxed. It should be noted that oligonucleotide design can only be as good as the underlying gene models, which, even for *Arabidopsis thaliana*, will continue to be updated for the foreseeable future. On the other hand, microarrays do provide a window of analysis of extraordinarily

Copyright © 2003 John Wiley & Sons, Ltd. 

high dimensionality (the Qiagen-Operon oligonucleotide set comprises 26 090 different sequences), and this feature can be used for classification purposes, even in the face of changing gene models. The importance of selecting non-redundant (i.e. non-cross-hybridizing) oligonucleotide sequences has recently been noted [37], and one should also be aware of the possibility of discordant measurements between amplicon- and oligonucleotide-based microarrays as a consequence of alternative splicing.

We have found that microarrays based on long oligomers appear to outperform their amplicon counterparts. Figure 2 illustrates an analysis of RNA extracted from *Arabidopsis* seedlings and hybridized to two separate microarrays, which were then compared by applying linear regression analysis to the replicate raw fluorescence intensity values. Considerable differences in performance were observed between the different platforms. Whereas the intensity values were highly correlated for the long-oligomer microarrays ($r^2 \sim 0.95$), those for amplicon microarrays were lower ($r^2 \sim 0.6$). Long oligomer arrays also displayed very low background fluorescence, an excellent dynamic range (3.5 decades) and the ability to detect most of the genes represented in the *Arabidopsis* genome [less than 5% were deemed undetectable, i.e. provided a signal intensity less than twice background (a signal of about 100 fluorescence units), under conditions of scanning that resulted in a saturated signal (65 536 FUs) from <0.5% of the array elements]. Figure 3 illustrates raw signals obtained from 102 genes classified as MYB domain-containing transcription factors. Using the same empirical criterion for detectability of expression excludes only five of these genes.

For expression analysis, an alternative to spotted microarrays is the use of surfaces on which DNA oligomers are synthesized, first commercialized by Affymetrix in the form of GeneChips™. The array elements comprise short (<25-mer) oligonucleotides, the monomers of which are sequentially added to the surface by covalent chemical reaction, using photolithographic techniques to deprotect specific spatial locations [29]. Design and construction of the series of photolithographic masks used for the deprotection steps is both costly and time-consuming. These input costs must be amortized across a substantial number of arrays to reduce costs to affordable levels. The requirement for mask construction also limits the flexibility of the platform. Any changes to the sequences synthesized on the array surface cannot be altered 'on the fly', since new masks must be designed and built to implement these changes.

This problem has recently been solved by developments pioneered at the University of Wisconsin [28,33] and commercialized by NimbleGen. NimbleGen chips are similar to Affymetrix GeneChips in that they comprise short sequences synthesized *in situ*. However, deprotection is achieved not by physical masks, but by using programmable micromirrors (Texas Instruments) to guide UV light to the precise surface locations at which deprotection is required. The primary advantage of NimbleGen technology over that of Affymetrix is the

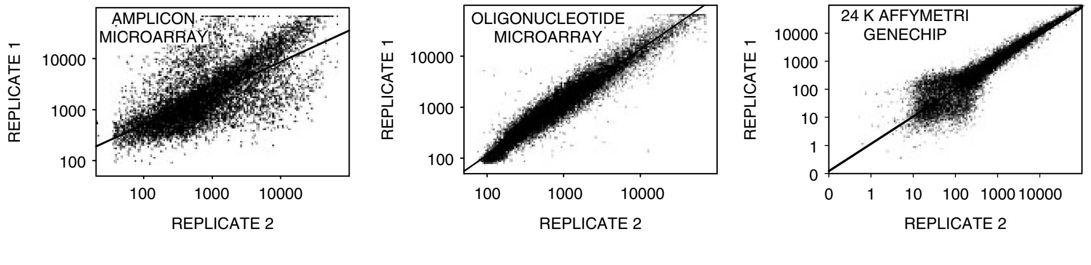

**Figure 2.** Reproducibility measurements comparing three expression platforms. In all cases, replicate RNA samples from Arabidopsis plants grown under similar conditions to similar growth stages were converted into targets, and were hybridized to two separate microarrays or GeneChips. Cy3 and Cy5 dye reversals were done for the microarrays. The intensity values for the corresponding array elements were then compared over the two replicates. In all cases, a line of correlation can be seen. Left panel: amplicon microarrays characteristically display a proportion of elements whose reproducibility is poor. Typically, these elements would be flagged and their contributions to technical replications ignored [10]. Centre panel: 70-mer oligonucleotide-based microarrays display remarkable reproducibility over the entire dynamic range. Right panel: for the GeneChips, good reproducibility is observed only for the upper 50% of the elements. Most of the genes in the lower half are called as 'Absent', and their reproducibilities are very low

Copyright © 2003 John Wiley & Sons, Ltd. 

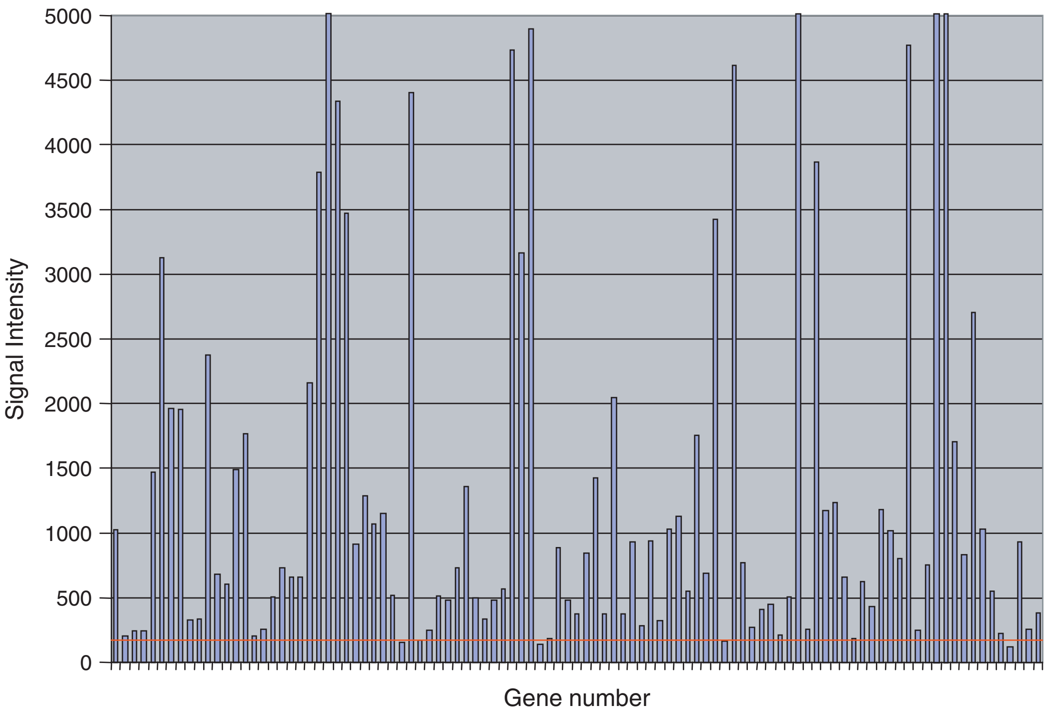

**Figure 3.** Raw signal intensity values obtained for 102 genes annotated as MYB domain-containing transcription factors. Only four of the signals were less than twice the local background (this value is indicated by the red line). Four of the array elements produced signals that were off-scale flexibility and low cost for redesign and production of modified arrays.

GeneChips have advantages and disadvantages relative to spotted microarrays. As for long-oligomer microarrays, array elements can be designed to maximize specificity. However, the shorter lengths of the array elements provide greater flexibility in this choice, which therefore suggests that GeneChips have the potential to be intrinsically more specific. This flexibility also, in theory, allows identification of specific oligomers that are particularly sensitive in transcript detection, whereas for long oligomers one is essentially restricted to one or at most a few different choices of sequence, none of which may be optimal for detection. One drawback of oligonucleotides synthesized *in situ* is that the efficiency of nucleotide incorporation per synthesis cycle (range 96–99%, dependent upon the type of chemistry employed) limits the final lengths of the oligonucleotides. For GeneChips, less than 5% of the sequences at any one location are full-length, others being shorter, capped, versions of the design sequence. Consequently, the hybridization events are close to the limits of duplex stabilization governed by thermodynamic considerations. For this reason, each gene sequence is represented by up to 20 different 25-mers at different GeneChip coordinates, hybridization being estimated based on the combined fluorescent signals from these probes. Signal specificity can also be calculated based on differences between 'perfect-match' (PM) and 'mismatch' (MM) probe sets, the MM probes being identical to their PM counterparts but with a single mismatched base at the central position of the sequence. The idea here is to eliminate contributions due to 'non-specific cross-hybridization', although the theory behind this approach does not appear particularly well founded and may have empirical flaws [9]. This approach also reduces the chip area available for different gene sequences by 50%, and some users exclusively employ GeneChips lacking MM sets for this reason. GeneChips are queried using single sets of fluorescent targets, rather than Cy3/Cy5 pairs as done for conventional microarrays, and this has the effect of doubling the numbers of

Copyright © 2003 John Wiley & Sons, Ltd.

chips required for the commonly employed pairwise comparisons of expression.

Analysis of the reproducibility of typical measurements obtained using GeneChips is also illustrated in Figure 2. Whereas for high fluorescence intensity measurements, the between-Chip correlations are excellent ($r^2 > 0.94$), at lower intensity values the correlations drastically decrease. This is evident in the characteristic broadening seen at the left-hand end of the distributions, commonly observed in other published datasets [7]. It is also reflected in the qualitative calls made by the Affymetrix data-analysis software, which define genes as Present, Marginal or Absent, based on the characteristics of the 20 pairs of intensity measurements made for each gene. For the data presented in the right-hand panel of Figure 3, produced using standard conditions of RNA extraction and labelling from *Arabidopsis* plants, ∼50% of the genes were called as Absent, and even for those genes called as Present, those of low signal intensity (i.e. those within the lower two quartiles of the datapoints) were poorly correlated ($r^2 \sim 0.15$).

Given the available platforms for analysis of gene expression, an emerging issue concerns the numbers of replications required for identification of statistically significant changes in mRNA levels. It is insufficient to apply criteria of fold-changes to identify genes of interest, simply because this does not address the problems of variation inherent to the individual genes that are being measured. We subscribe to the view proposed by Kerr and Churchill [23] and Wolfinger *et al.* [38], among others, that microarray experiments should be designed, and the datasets produced and rigorously evaluated, using statistical methods, particularly analysis of variance (ANOVA). ANOVA requires a sufficient number of replicate arrays for data generation, and can only be possible for expression platforms of relatively low cost. For the spotted microarrays, we routinely employ a two-stage linear ANOVA: first, global variation is evaluated across the arrays as a function of dye type and individual RNA samples. The unexplained variance, corresponding to the gene-by-treatment changes of experimental interest, provides the input to the second stage to determine whether these changes are statistically significant. Based on this analysis, the long oligo arrays in our hands show essentially no background variance (<0.5% of the total variance) and no evidence of dye-specific or microarray positional effects. With the given number of replications, gene expression changes are then categorized as significant or non-significant. A recent paper has discussed the application of ANOVA to GeneChip experiments [9]. Their conclusions were that: (a) additional statistical power is obtained if all data values are employed, rather than the averages of the individual hybridization values for the different 25-mers representing individual genes; (b) direct subtraction of MM from PM values has no advantage and, in fact, only adds noise to the dataset; and (c) replication is essential to bring out the full power of the GeneChips.

The importance of replicating microarray/GeneChip experiments cannot be understated. The high technical reproducibility of the long oligomer arrays contrasts very favourably with other platforms, suggesting the number of required technical replicates will be fewer than for other platforms. However, replication is always required to address biological variability, and sufficient data is needed to determine whether given changes in transcript level are, on a gene-by-gene basis, statistically significant. Fiscal issues limit replications of microarray and GeneChip experiments. In terms of cost, long oligonucleotide microarrays outperform GeneChips by a factor of 8 (*Arabidopsis* GeneChips cost ∼\$400 each, whereas long oligo arrays at ∼\$100 each produce twice as many data points).

### Identification and purification of different cell types

Developing methods for analysis of specific cell types requires means for identification of these cell types that are compatible with methods for their subsequent purification. At the molecular level, the most flexible way to identify different cell types relies on the observation that specific genes are frequently found that are uniquely transcribed within these cell types. Transgenic gene technologies can then be employed to specifically highlight these cells via expression of heterologous markers. Particularly suitable for the purification step is the use of fluorescent proteins (FPs) as markers, a class of proteins for which the green fluorescent protein (GFP) of *Aequorea victoria* is the founding member [6]. Fluorescent cells can be conveniently purified from their non-fluorescent counterparts by fluorescence-activated cell sorting (FACS).

Copyright © 2003 John Wiley & Sons, Ltd. 

One prerequisite for successful use of FACS is that the input population comprises a single-cell suspension. This is because each cell must be individually queried as it passes in a constrained fluid stream through the focus of an intense light source, and then sorted following its emergence from the flow cell tip. A second prerequisite is that the cells be optically homogeneous, i.e. as far as possible they should be spherical, with little variation in diameters across the population, and without unusual light scattering or endogenous fluorescence or absorbance characteristics. Sorting large cells (>50 μm in diameter) is also technically demanding [13], and therefore less desirable. Methods for flow cytometric analysis of plant protoplasts and animal cell populations based on FP expression are well established [14,16]. Recent progress has included multicolor FP flow analysis and sorting [21], as well as examples of the sorting of a variety of different cell types from different organisms [11,30,34].

The major problem confronting the application of flow cytometric techniques for sorting specific cell types concerns the requirement for single-cell suspensions. For mammals, some tissues exist as natural single-cell suspensions, e.g. the circulating cells of the haematopoietic system, and cell cultures in general. The cells of animal organs, on the other hand, exist as complex three-dimensional interspersions. Producing single-cell suspensions from these organs requires the use of proteolytic enzymes to hydrolyse the proteins and glycoproteins mediating specific cell–cell interactions. Similar problems of tissue architecture are encountered in considering plant organs. In this case, conversion of plant tissues to protoplasts, using wall-degrading enzymes, provides the means for production of single cell suspensions. An addition layer of complexity is a consequence of the presence of a hypertonic osmoticum during cell wall hydrolysis, required to prevent plasma membrane lysis. It appears inevitable that global patterns of gene expression will be perturbed by alterations to intercellular communication and, in the latter case, by osmotic stress, based on what is already known about the behaviour of organisms subjected to these treatments. This question has been addressed by several groups, including our own [19,32]. Surprisingly, perhaps, it appears that protoplasts maintain many features of normal cellular regulation, at least for a limited period of time. During this period, transfection methods can be employed to probe signal transduction pathways using FP reporters [32]. A further complication in plant protoplast preparation is the accessibility of specific cell types, and the susceptibilities of their walls to enzymatic hydrolysis. Protoplast preparation is also not downwardly scalable in terms of cell number (due to non-specific adhesion of protoplasts to glass and plastic-ware, amongst other issues) and below a certain protoplast yield, recovery of viable protoplasts is very difficult to achieve.

### Flow analysis and sorting of nuclei based on FP expression

Given that the process of protoplast production has the potential to perturb gene expression and that protoplast preparation and sorting is technically difficult, we have proposed an alternative approach to analysis of cell and tissue-specific gene expression that simultaneously solves both problems. This is based on the observation that flow analysis and sorting can be done using tissue homogenates, assuming that a fluorescent signal can be specifically associated with the objects to be sorted. Given the role of the nucleus in transcript production (Figure 1), we have based our strategy on the idea of flow analysis and sorting as a means to specifically purify nuclei labelled through directed targeting of FP-fusions. Flow analysis of nuclei within tissue homogenates is a robust procedure devised originally for characterization of genome sizes, ploidy status and the activity of the cell cycle [15]. We have also established that flow analysis and sorting can also be employed for detection of GFP within the nuclei of transgenic tobacco [17]. The large size of the tobacco genome, and hence that of the nucleus, relative to those of many other plant species, facilitates the detection of the GFP-specific signal, which is derived from nuclear localization signal-mediated accumulation of a chimeric GFP-$\beta$-glucuronidase protein over that of other fluorescent particles in the homogenate. In *Arabidopsis thaliana*, which has a much smaller genome, it appears that fusion of GFP to nuclear structural proteins (such as histones) provides a signal that is more readily detected above this background (Zhang, Lambert and Galbraith, unpublished).

Copyright © 2003 John Wiley & Sons, Ltd. 

### Highlighting specific cell types through FP expression and targeting

Essential to the concept of employing flow cytometric methods for purifying single cells or their nuclei for analysis of specific gene expression is the ability to readily identify gene sequences that regulate FP expression within specific cell types of interest. A variety of promoters are known to exhibit tissue-specific patterns of expression in higher plants (see e.g. [27]), and various projects are under way around the world that are aimed to systematically describe DNA sequences that regulate expression in a tissue- or cell type-specific manner. These projects essentially involve high-throughput FP-based promoter and enhancer trapping, the most advanced of which has produced a number of *Arabidopsis* lines exhibiting cell type-specific FP expression [20]. Adapting these lines for expression of nuclear-targeted FP is facilitated by their use of the GAL4-UAS/DBD system to drive marker gene expression.

### Combining technologies for global analysis of cell type-specific gene expression

Flow sorting of FP-tagged protoplasts or nuclei can be done at high rates. Although sorting of protoplasts requires large flow tips (100 μm or greater), which reduces the rates of sorting due to physical considerations governing the process of droplet break-off [18], we routinely sort protoplasts at 50–1000 positive events/s. Nuclei, due to their small sizes, can be sorted using smaller flow tips (50 μm in diameter), which allows operation of the Cytomation MoFlo flow cytometer at its upper sort rate limit (an event rate of ∼70 000/s). Thus, sorting is limited only by source materials, e.g. if one were interested in a specific cell type that is present at only one or two cells/plant. Under these circumstances, amplification of the RNA signal, prior to microarray analysis, becomes essential. Various methods for target amplification have been reported (for a recent discussion, see [12]). Of these, linear amplification methods, rather than those based on PCR, appear to be most appropriate, since they are likely to introduce less distortion into the patterns of expression that are subsequently measured. Amplification has been reported to increase the reproducibility of ratio measurements of amplicon-based microarrays [12], and it would be of interest to see whether this is also true of the raw intensity measurements.

### Conclusions and prospects

The last decade has seen extraordinary advances in our ability to chart global gene expression. The combinations of technologies outlined in this review are not restricted by species, or even to particular kingdoms. They should permit a detailed description, at the level of individual cells, of the processes of normal development, as well as those underlying diseased states, and responses to environmental changes. It should be noted that these methods should also be applicable to the global study of non-coding RNAs, for which important roles in gene regulation, as well as tissue-specificity of expression, have recently been uncovered [24].

### Acknowledgements

I thank Michael Thomashow and Sarah Palmer (MSU) for providing the Affymetrix data, and Rangasamy Elumalai (Arizona) and Michael Deyholos (Edmonton) for the microarray data represented in Figure 2. I thank former and current members of the Galbraith laboratory and other associates at the University of Arizona for helpful discussions, particularly M. Deyholos, R. Elumalai, G. Lambert, M. Nouzova, C. Vanier, H. Wang and C. Zhang. This work was supported by grants DBI 9872657, 9813360 and 0211857 from the NSF Plant Genome Research Project, and is dedicated to the memory of David C. Rowe.

### References

1. Bachem CWB, van der Hoeven RS, de Bruijn SM, *et al.* 1996. Visualization of differential gene expression using a novel method of RNA fingerprinting based on AFLP: analysis of gene expression during potato tuber development. *Plant J* **95**: 745–753.
2. Brenner S. 2003. The way ahead. Plenary presentation, Plant and Animal Genome XIth Conference, San Diego, CA.
3. Brenner S, Williams SR, Vermaas EH, *et al.* 2000. *In vitro* cloning of complex mixtures of DNA on microbeads: physical separation of differentially expressed cDNAs. *Proc Natl Acad Sci USA* **97**: 1665–1670.
4. Brenner S, Johnson M, Bridgham J, *et al.* 2000. Gene expression analysis by massively parallel signature sequencing (MPSS) on microbead arrays. *Nature Biotechnol* **18**: 630–634.

Copyright © 2003 John Wiley & Sons, Ltd. 

5. Brown V, Jin P, Ceman S, *et al*. 2001. Microarray identification of FMRP-associated brain mRNAs and altered mRNA translational profiles in fragile X syndrome. *Cell* **107**: 477–487.
6. Chalfie M, Tu Y, Euskirchen G, Ward WW, Prasher DC. 1994. Green fluorescent protein as a marker for gene expression. *Science* **263**: 802–805.
7. Che P, Gingerich DJ, Lall S, Howell SH. 2002. Global and hormone-induced gene expression changes during shoot development in *Arabidopsis*. *Plant Cell* **14**: 2771–2785.
8. Cho Y, Fernandes J, Kim SH, Walbot V. 2002. Gene-expression profile comparisons distinguish seven organs of maize. *Genome Biol* **3**: research 0045.1–0045.16.
9. Chu TM, Weir B, Wolfinger R. 2002. A systematic statistical linear modeling approach to oligonucleotide array experiments. *Math Biosci* **176**: 35–51.
10. Deyholos MK, Galbraith DW. 2001. High-density DNA microarrays for gene expression analysis. *Cytometry* **43**: 229–238.
11. Fei YJ, Hughes TE. 2001. Transgenic expression of the jellyfish green fluorescent protein in the cone photoreceptors of the mouse. *Visual Neurosci* **18**: 615–623.
12. Feldman AL, Costouros NG, Wang E, *et al*. 2002. Advantages of mRNA amplification for microarray analysis. *Biotechniques* **33**: 906–914.
13. Galbraith DW, Lucretti S. 2000. Large particle sorting. In *Flow Cytometry and Cell Sorting*, 2nd edn, Radbruch A (ed.). Springer-Verlag: Berlin; 293–317.
14. Galbraith DW, Anderson MT, Herzenberg LA. 1998. Flow cytometric analysis and sorting of cells based on GFP accumulation. *Methods Cell Biol* **58**: 315–341.
15. Galbraith DW, Harkins KR, Maddox JR, *et al*. 1983. Rapid flow cytometric analysis of the cell cycle in intact plant tissues. *Science* **220**: 1049–1051.
16. Galbraith DW, Herzenberg LA, Anderson M. 1999. Flow cytometric analysis of transgene expression in higher plants: green fluorescent protein. *Methods Enzymol* **320**: 296–315.
17. Grebenok RJ, Lambert GM, Galbraith DW. 1997. Characterization of the targeted nuclear accumulation of GFP within the cells of transgenic plants. *Plant J* **12**: 685–696.
18. Harkins KR, Galbraith DW. 1987. Factors governing the flow cytometric analysis and sorting of large biological particles. *Cytometry* **8**: 60–71.
19. Harkins KR, Jefferson RA, Kavanagh TA, Bevan MW, Galbraith DW. 1990. Expression of photosynthesis-related gene fusions is restricted by cell-type in transgenic plants and in transfected protoplasts. *Proc Natl Acad Sci USA* **87**: 816–820.
20. Haseloff J. 1999. GFP variants for multispectral imaging of living cells. *Methods Cell Biol* **58**: 139–151.
21. Hawley TS, Telford WG, Ramezani A, Hawley RG. 2001. Four-color flow cytometric detection of retrovirally expressed red, yellow, green and cyan fluorescent proteins. *Biotechniques* **30**: 1028–1035.
22. Johannes G, Carter MS, Eisen MB, Brown PO, Sarnow P. 1999. Identification of eukaryotic mRNAs that are translated at reduced cap binding complex eIF4F concentrations using a cDNA microarray. *Proc Natl Acad Sci USA* **96**: 13 118–13 123.
23. Kerr MK, Martin M, Churchill GA. 2000. Analysis of variance for gene expression microarray data. *J Comp Biol* **7**: 819–837.
24. Lagos-Quintana M, Rauhut R, Yalcin A, *et al*. 2002. Identification of tissue-specific microRNAs from mouse. *Curr Biol* **12**: 735–739.
25. Macas J, Lambert GM, Dolezel D, Galbraith DW. 1998. NEST (nuclear expressed sequence tag) analysis: a novel means to study transcription through amplification of nuclear *RNA*. *Cytometry* **33**: 460–468.
26. Martel RR, Botros IW, Rounseville MP, *et al*. 2002. Multiplexed screening assay for mRNA combining nuclease protection with luminescent array detection. *Assay Drug Dev Technol* **1**: 61–71.
27. Nakajima K, Sena G, Nawy T, Benfey PN. 2001. Intercellular movement of the putative transcription factor SHR in root patterning. *Nature* **413**: 307–311.
28. Nuwaysir EF, Huang W, Albert TJ, *et al*. 2002. Gene expression analysis using oligonucleotide arrays produced by maskless photolithography. *Genome Res* **12**: 1749–1755.
29. Pease AC, Solas D, Sullivan EJ, *et al*. 1994. Light-generated oligonucleotide arrays for rapid DNA sequence analysis. *Proc Natl Acad Sci USA* **91**: 5022–5026.
30. Sawamoto K, Yamamoto A, Kawaguchi A, *et al*. 2001. Direct isolation of committed neuronal progenitor cells from transgenic mice co-expressing spectrally distinct fluorescent proteins regulated by stage-specific neural promoters. *J Neurosci Res* **65**: 220–227.
31. Schena M, Shalon D, Davis RW, Brown PO. 1995. Quantitative monitoring of gene expression patterns with a complementary DNA microarray. *Science* **270**: 467–470.
32. Sheen J. 2001. Signal transduction in maize and *Arabidopsis* mesophyll protoplasts. *Plant Physiol* **127**: 1466–1475.
33. Singh-Gasson S, Green RD, Yue Y, *et al*. 1999. Maskless fabrication of light-directed oligonucleotide microarrays using a digital micromirror array. *Nature Biotechnol* **17**: 974–978.
34. Takeuchi Y, Yoshizaki G, Kobayashi T, Takeuchi T. 2002. Mass isolation of primordial germ cells from transgenic rainbow trout carrying the green fluorescent protein gene driven by the vasa gene promoter. *Biol Reprod* **67**: 1087–1092.
35. Velculescu VE, Zhang L, Vogelstein B, Kinzler KW. 1995. Serial analysis of gene expression. *Science* **270**: 484–487.
36. Vos P, Hogers R, Bleeker M, *et al*. 1995. AFLP — a new technique for DNA fingerprinting. *Nucleic Acids Res* **23**: 4407–4414.
37. Wang H-Y, Malek RL, Anne E, *et al*. 2003. Assessing unmodified 70-mer oligonucleotide probe performance on glass-slide microarrays. *Genome Biol* **4**: R5.1–13.
38. Wolfinger RD, Gibson G, Wolfinger ED, *et al*. 2001. Assessing gene significance from cDNA microarray expression data via mixed models. *J Comput Biol* **8**: 625–637.
39. Xu W, Bak S, Decker A, *et al*. 2001. Microarray-based analysis of gene expression in very large gene families: the cytochrome P450 gene superfamily of *Arabidopsis thaliana*. *Gene* **272**: 61–74.

Copyright © 2003 John Wiley & Sons, Ltd. 
